# A fast approach to determine a fed batch feeding profile for recombinant *Pichia pastoris *strains

**DOI:** 10.1186/1475-2859-10-85

**Published:** 2011-10-27

**Authors:** Christian Dietzsch, Oliver Spadiut, Christoph Herwig

**Affiliations:** 1 Vienna University of Technology, Institute of Chemical Engineering, Research Area Biochemical Engineering, Vienna, Austria

**Keywords:** *Pichia pastoris*, strain characterization, specific substrate uptake rate, batch cultivation, methanol pulse, dynamic feeding profile

## Abstract

**Background:**

The microorganism *Pichia pastoris *is a commonly used microbial host for the expression of recombinant proteins in biotechnology and biopharmaceutical industry. To speed up process development, a fast methodology to determine strain characteristic parameters, which are needed to subsequently set up fed batch feeding profiles, is required.

**Results:**

Here, we show the general applicability of a novel approach to quantify a certain minimal set of bioprocess-relevant parameters, *i.e*. the adaptation time of the culture to methanol, the specific substrate uptake rate during the adaptation phase and the maximum specific substrate uptake rate, based on fast and easy-to-do batch cultivations with repeated methanol pulses in a batch culture. A detailed analysis of the adaptation of different *P. pastoris *strains to methanol was conducted and revealed that each strain showed very different characteristics during adaptation, illustrating the need of individual screenings for an optimal parameter definition during this phase. Based on the results obtained in batch cultivations, dynamic feeding profiles based on the specific substrate uptake rate were employed for different *P. pastoris *strains. In these experiments the maximum specific substrate uptake rate, which had been defined in batch experiments, also represented the upper limit of methanol uptake, underlining the validity of the determined process-relevant parameters and the overall experimental strategy.

**Conclusion:**

In this study, we show that a fast approach to determine a minimal set of strain characteristic parameters based on easy-to-do batch cultivations with methanol pulses is generally applicable for different *P. pastoris *strains and that dynamic fed batch strategies can be designed on the specific substrate uptake rate without running the risk of methanol accumulation.

## Background

Advances in molecular biology, cloning techniques and strain improvement allowed an increasing use of recombinant organisms for the industrial production of a variety of substances like organic acids, antibiotics, enzymes and amino acids. In this context the methylotrophic yeast *Pichia pastoris *is one of the most important host organisms for the expression of recombinant proteins. To meet industrial demands, a fast and easy-to-do characterization of recombinant *Pichia *strains to extract bioprocess-relevant strain characteristic parameters for the subsequent set-up of production processes is essential to speed up process development. Normally, this strain characterization procedure is done by time-consuming experiments, which require complex and costly equipment, like continuous cultures [1,2] or several, consecutive fed batch cultivations, operated at different conditions [3,4]. Parameters, which have to be extracted out of these experiments, describe the best operating conditions for each strain as well as the optimal condition for the adaptation of the culture from the growth substrate (e.g. glucose or glycerol) to the inducer methanol. To date, different strategies are employed regarding the adaptation of *Pichia *to methanol; two prominent examples are: 1) after a fed batch on glycerol or glucose a certain low concentration or flow of methanol is applied to the culture which is then increased to a pre-defined maximum and constantly maintained throughout the whole cultivation time [5,6], and 2) the glycerol flow in the reactor is decreased following a linear function during a concomitant addition of methanol, a period which is called transition phase, to slowly adapt the culture to methanol [7-10]. These methods are often based on specific experiences with a certain strain, but are nevertheless often used as a general approach for different *Pichia *strains in following studies, without taking into account the specific requirements of the single strains during adaptation.

After adaptation of the culture to methanol, different feeding strategies can be employed for recombinant protein production with *P. pastoris*. Besides the two common strategies of either using a feed forward regime or a controlled specific growth rate (μ) [6,7,9-14], a few studies have also described the importance of the specific substrate uptake rate (q_s_) on recombinant protein production [15,16]. A direct correlation between q_s _and the specific productivity (q_p_) was shown [15,16], and it was clearly stated, that q_s _was the most important induction parameter in these experiments [17]. Based on those findings and motivated by problems which occur, when more traditional feeding strategies are applied (e.g. possible accumulation of methanol caused by changing cell capacities during cultivation or the need of expensive monitoring equipment to allow μ-controlled feeding [9,14]), we have focused our research on the specific substrate uptake rate (q_s_) and have recently shown optimization potential using dynamic feeding profiles based on this parameter [18].

In our previous study we also developed a fast approach based on batch experiments with methanol pulses to extract a minimal set of strain characteristic parameters (*i.e*. Δtime_adapt _- time for adaptation, q_s adapt _- specific substrate uptake rate during adaptation, q_s max _- maximum specific substrate uptake rate), which are required to set up a subsequent feeding regime based on q_s_. However, our previous study dealt with the development and the application of this approach for only one recombinant *P. pastoris *Mut^S ^strain [18]. In the present work, we characterized various *P. pastoris *strains with different phenotypes (Mut^s ^and Mut^+^) expressing different target enzymes using the above mentioned strategy. We analyzed the required time for adaptation to methanol of each strain in detail and could reliably derive certain strain characteristic parameters from pulse experiments to fed batch cultivations. With the variety of used strains in this study, we demonstrate that this approach is generally applicable for different *P. pastoris *strains and is thus a valuable tool for fast process development, which is especially interesting in an industrial environment.

## Materials and methods

The experiments conducted in the present study were performed according to our previous study [18], and are thus only described briefly here.

### Microorganisms and recombinant proteins

Different *P. pastoris *strains with different phenotypes expressing different target enzymes were used in this study to prove the general applicability of our strategy. A list of the various strains is given in Table 1.

**Table 1:** Different *P. pastoris *strains used in this study

Strain	phenotype	expressed enzyme	in this study designated as
KM71H	Mut^S^	-	KM71H
KM71H	Mut^S^	HRP	KM71H HRP
KM71H	Mut^S^	PDI and HRP	KM71H PDI HRP
CBS7435	Mut^S^	HRP	CBS7435 HRP
SMD1168H	Mut^+^	GalOX	SMD1168H GalOX

All recombinant genes used in this study were under the control of the AOX1 promoter. The expressed HRP gene coded for the isoenzyme HRP C1A. The strain KM71H PDI HRP concomitantly expressed HRP and the chaperone protein disulfide isomerase (PDI), which was under the control of a modified AOX1 promoter [19]. The strains KM71H, KM71H HRP, KM71H PDI HRP and CBS7435 HRP were gratefully provided by Prof. Anton Glieder (Graz University of Technology, Austria). The strain SMD1168H GalOX was constructed by Spadiut *et al*., as described elsewhere [20].

### Culture Media

Precultures were performed in complex yeast nitrogen base media (YNBM), whereas batch and fed batch cultivations were done in defined basal salt media (BSM; [21]). The glucose feed was prepared with glucose (250 g·l^-1^), trace element solution PTM1 (12 ml·l^-1^) and antifoam Struktol J650 (0.3 ml/l). The methanol feed was composed of methanol (300 g·l^-1^), PTM1 (4 ml·l^-1^) and Struktol J650 (0.3 ml·l^-1^). The induction period for HRP expression was carried out in the presence of δ-Aminolevulinic acid (δ-ALA) in a final concentration of 1 mM. The concentration of the base NH_4_OH was determined by titration with 0.25 M potassium hydrogen phthalate (KHP).

### Experimental Procedure

#### Preculture

Frozen stocks (-80°C) were precultivated in 100 ml of YNBM in 1000 ml shake flasks at 28°C and 230 rpm for max. 24 hours.

#### Batch cultivation with methanol pulses

Batch cultivations were carried out in a 5 l working volume glass bioreactor (Infors, Switzerland) at 28°C and a fixed agitation speed of 1200 rpm. The culture was aerated with 1 vvm dried air and off-gas was measured by using an infrared cell for CO_2 _and a paramagnetic cell for O_2 _concentration (Servomex, Switzerland). Process parameters were recorded and logged in a process information management system (PIMS; Lucullus, Biospectra, Switzerland). After the complete consumption of glucose, which was indicated by an increase of dissolved oxygen and a drop in off-gas activity, the first methanol pulse (adaptation pulse) with a final concentration of 0.5% (v/v) was conducted with pure methanol (supplemented with PTM1, 12 ml·l^-1 ^of methanol). Following pulses were performed with 1% (v/v) concentration of methanol. For all strains, several pulses were conducted after the adaptation pulse to generate consistent data for each strain. For each methanol-pulse, at least two samples were taken to determine the concentrations of substrate and product as well as dry cell weight and OD_600 _to calculate the specific substrate uptake rate q_s_.

#### Fed batch cultivations

Fed batch cultivations were carried out in a 5 l working volume glass bioreactor (Infors, Switzerland) in 2-fold concentrated BSM medium at 28°C and 1500 rpm. The culture was aerated with at least 1 vvm to keep dissolved oxygen levels > 30%. In case, air flow was limited, pure oxygen was added. The fed batch feed was measured and controlled using a gravimetrically based PID flow controller. At several time points during fed batch cultivations, samples were taken and analyzed for accumulated methanol, biomass concentration (dry cell weight and optical density OD_600_) and, if applicable, enzymatic activity. Based on the total biomass content, feeding rates were adjusted manually corresponding to the defined q_s _set point. All fed batches described in this study were conducted in the same way: after an adaptation period at q_s _of 0.5 mmol·g^-1^·h^-1^, a stepwise increase of q_s _up to q_s max _of the respective strain was carried out with step times of 24 hours.

### Analysis of growth- and expression-parameters

Dry cell weight (DCW), OD_600_, substrate concentrations as well as the catalytic activity of HRP were determined as described before [18]. However, in this study the ABTS solution for HRP activity measurements was prepared in 50 mM KH_2_PO_4_-buffer at pH 6.5 and the calibration range was expanded to 2.0 U·ml^-1^. Also GalOX activity was measured with an ABTS assay; *i.e*. a sample of diluted enzyme (10 μl) was added to 990 μl of assay buffer containing horseradish peroxidase (222 U) (Type VI-A, Sigma-Aldrich, P6782), ABTS (17.7 mg), KH_2_PO_4_-buffer (50 mM, pH 6.5) and D-galactose (300 mM). The absorbance change at 420 nm (ε_420 _= 42.3 mM^-1^·cm^-1^) was recorded at 30°C for 180 seconds. One Unit of GalOX activity was defined as the amount of enzyme necessary for the oxidation of 2 μmol of ABTS per min, corresponding to the consumption of 1 μmol of O_2 _per min. An additional post-translational activation of GalOX by adding CuSO_4 _to the samples in the presence of oxygen before activity measurements, as described elsewhere [20], was not executed.

### Specific rate calculations

#### Batch cultivations

To obtain the specific rates for substrate uptake and productivity, samples were taken at certain time points during the methanol pulses (*i.e*. beginning of pulse, maximum off-gas of pulse, end of pulse) and analyzed offline for biomass content, methanol concentration and, if applicable, enzymatic activity. Determined values at the beginning and the end of the respective pulse were used to calculate an average rate of the specific substrate uptake, which was corrected for stripping using Antoine's equation, and the specific productivity. Errors for specific rates were set to 10%, according to our previous study [18]. Online calculated carbon dioxide evolution rate (CER) was divided by actual biomass concentrations to obtain the specific carbon dioxide production rate (qCO_2_). In addition, a time derivative of the qCO_2 _signal (*i.e*. qCO_2_') was calculated using a time window of 30 minutes (15 minutes before and 15 minutes after the actual time point).

#### Fed batch cultures

During different cultivation periods, representing defined q_s _set points, several samples were taken and OD_600 _measurements were used to calculate the actual total biomass content, which allowed adjustments of the methanol feed flow to the actual q_s _set point. Specific rates were calculated using DCW and the amount of consumed methanol, which was determined gravimetrically. Presented results correspond to an average value over the respective q_s _set point period. Again, errors for specific rates were set to 10%.

## Results and Discussion

### A fast approach to derive a minimal set of strain characteristic parameters relevant for bioprocess development

Each *P. pastoris *strain was cultivated in an easy-to-do batch system with methanol pulses to obtain certain strain characteristic parameters during the adaptation period (Δtime_adapt _- time for adaptation of the culture to methanol, q_s adapt _- specific substrate uptake rate during the adaptation pulse) and the maximum specific substrate uptake rate (q_s max_). These parameters were extracted and consecutively transformed into a feeding profile for fed batch operations based on q_s_.

#### Adaptation of the culture to methanol

**Figure 1:**
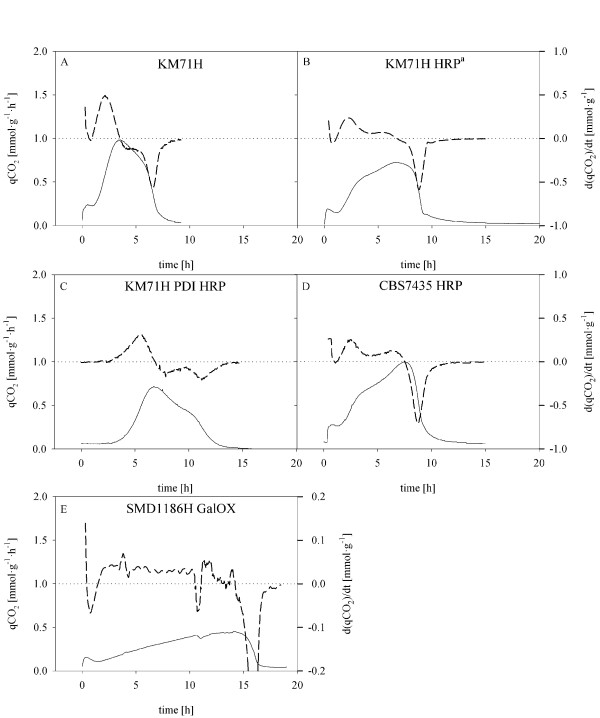
**Adaptation pulses with 0.5% (v/v) methanol after glucose depletion for different *P. pastoris *strains**. Straight line, specific carbon dioxide production rate qCO_2_; dashed line, time-derivative of the specific carbon dioxide production rate qCO_2_' (d(qCO_2_)/dt); A, KM71H; B, KM71H HRP^a^; C, KM71H PDI HRP; D, CBS7435 HRP; E, SMD1168H GalOX. ^a^data taken from [18]

After depletion of glucose in batch cultivation, a methanol adaptation pulse with a final concentration of 0.5% (v/v) was applied. The time required to develop a maximum in off-gas activity was used to define Δtime_adapt_, according to our previous study [18], and is shown here as the specific carbon dioxide production rate (qCO_2_; Figure 1).

The used strains showed very different metabolic characteristics during the adaptation to methanol. The shortest Δtime_adapt _of 3.5 h was detected for the KM71H strain, which was a Mut^S ^strain, not carrying a recombinant gene for heterologous protein expression. The other Mut^S ^strains KM71H HRP, KM71H PDI HRP and CBS7435 HRP (Figure 1B, C and 1D, respectively) showed 2-fold longer adaptation times compared to the KM71H strain (see also Table 2). These results clearly show that recombinant *Pichia *strains, which heterologously produce proteins upon the presence of the inducer methanol, carry an additional biological burden which significantly changes their metabolism and slows down their adaptation to methanol.

**Table 2:** Batch experiments with methanol pulses to determine strain specific parameters of different *P. pastoris *strains.

	KM71H	KM71H HRP^a^	KM71H PDI HRP	CBS7435 HRP	SMD1168H GalOX
Δtime_adapt Batch _[h]	3.5	7	7	7.5	14

max. qCO_2_' [h]	2.1	2.3	5.7	2.5	2.7

q_s adapt_ [mmol·g^-1^·h^-1^]	0.96 ± 0.10	0.80 ± 0.08	0.56 ± 0.06	0.77 ± 0.08	0.48 ± 0.05

q_s max_^b^ [mmol·g^-1^·h^-1^]	1.94 ± 0.19	2.00 ± 0.20	1.08 ± 0.10	1.54 ± 0.15	2.62 ± 0.26

q_p max_^b^ [U·g^-1^·h^-1^]	-	2.5 ± 0.25	6.3 ± 0.63	4.25 ± 0.43	200.8 ± 20.1

^a ^data taken from [18]

^b ^representing the maximum value determined out of all pulses

Surprisingly, the *P. pastoris *Mut^+ ^strain SMD1168H GalOX showed the longest adaptation time of 14 h (Figure 1E). However, this maximum was just detected as a result of methanol depletion and thus Monod kinetics, rather than representing the real time point of full methanol adaptation. As shown in Figure 1, the Mut^+ ^strain SMD1168H GalOX showed a very different metabolic behaviour with a flat slope for qCO_2 _compared to the qCO_2 _curves of the Mut^S ^strains, which might be due to intracellular regulation and inhibition events, caused by produced H_2_O_2 _and the absence of sufficient catalases in the cells. Regulation events like this have been described in various systems before [22-24]. Thus, the determined Δtime_adapt _of 14 h for the Mut^+ ^strain is questionable and the cells had probably been adapted to methanol before.

Consequently, the usefulness of the strain characteristic parameter Δtime_adapt_, especially with regard to the Mut^+ ^strain SMD1168H GalOX, was checked by introducing a time derivative of the qCO_2 _signal (qCO_2_'). Since qCO_2 _and the specific growth rate μ are linearly related, the maximum of qCO_2_' represents the time point of adaptation of the culture to the new substrate methanol. At the maximum qCO_2_' the cells should be already fit for further assimilation to methanol, and thus this point represents a good starting point for consecutive fed batch cultivations without running the risk of methanol accumulation. A good example for this is shown in Figure 1A for the strain KM71H, where a rising slope is shown with a single qCO_2_' maximum after 2.1 h. However, the other Mut^S ^strains tested (KM71H HRP, KM71H PDI HRP and CBS7435 HRP) were characterized by a more bumpy qCO_2 _curve, resulting in a qCO_2_' signal with several shoulders (Figure 1B, C and 1D, respectively). Apparently, the adaptation of these strains to methanol did not happen as straight-forward as for the strain KM71H, but with local minima and maxima of the metabolic capacity probably caused by regulatory events upon an excess of methanol. For the Mut^+ ^strain SMD1168H GalOX the specific carbon dioxide production was rather low compared to the Mut^S ^strains. The maximum in qCO_2_' was determined already after 2.7 h, which was by far sooner than the observed Δtime_adapt _of 14 h. The quite constant qCO_2_' signal over time (Figure 1E), further supports the fact that the cell capacity was already adapted to its maximum after this short time of 2.7 h and that the cells had been fully adapted to methanol much sooner than the Δtime_adapt _of 14 h.

**Figure 2:**
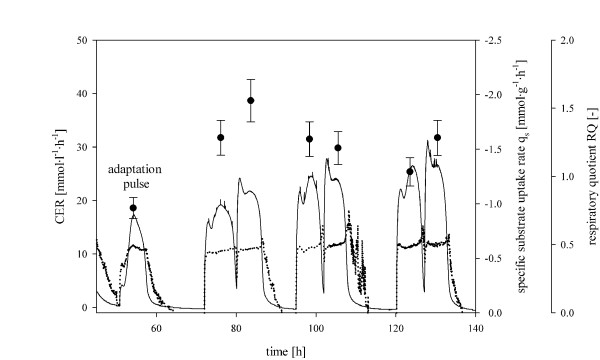
**Batch cultivation of the strain KM71H with a 0.5% (v/v) methanol pulse for adaptation and 1% (v/v) methanol pulses for q_s max _determination**. Straight line, calculated carbon dioxide evolution rate (CER); dotted line, respiratory quotient RQ (CER/OUR); black dot, specific substrate uptake rate (q_s_) for each pulse.

In general, the more detailed analysis of the different strains in their adaptation to methanol revealed three different patterns in the qCO_2_' signal: 1) with a single maximum, 2) with several shoulders and 3) a quite constant signal over time. This underlines the necessity for individual analyses of different strains in order to quantitatively characterize them during the adaptation phase in the presence of methanol excess. Of course, the observed maximum in off-gas activity is dependent on the affinity of the cells to the substrate methanol. The maximum in qCO_2 _could also be reached in terms of substrate limitation and a consequent drop in the qCO_2 _signal due to Monod kinetics rather than by the maximum metabolic adaptation to methanol, which in this study can clearly be seen for the Mut^+ ^strain SMD1186H GalOX (Figure 1E). However, for all strains tested in this study, maximum values of qCO_2_' were reached before the maximum off-gas activity (Figure 1 and Table 2), demonstrating that the applied concentration of methanol in the adaptation pulse was high enough to guarantee that the maximum qCO_2_' was reached independent of Monod kinetic effects. The validity of qCO_2_' as a reliable signal to detect the adaptation of the culture to methanol is further underlined when analyzing the respiratory quotient (RQ) during the adaptation pulse, which is exemplarily shown for the strain KM71H in Figure 2. During the adaptation pulse, RQ fluctuates with local minima and maxima until the signal becomes rather constant indicating the adaptation of the culture to methanol, which actually coincides with the maximum of qCO_2_'. Bespoken fluctuations of RQ at the beginning of the adaptation pulse represent the differences in catabolic and anabolic activity of the adapting cells. Similar effects have been observed for *Saccharomyces cerevisiae *[22,25,26]. During the following pulses, RQ shows a rather constant signal indicating that the cells had already been adapted to the new substrate. These findings validate the parameter qCO_2_' as a reliable indicator for methanol adaptation.

However, despite the advantage of describing the time point of adaptation of the culture to methanol more accurately, the use of qCO_2_' as a parameter to determine the starting point of the following fed batch could be risky because of the described fluctuations in qCO_2 _(Figure 1). On the other hand, the maximum off-gas activity (Δtime_adapt_) is a parameter which safely describes methanol adaptation. Another advantage of using Δtime_adapt _instead of qCO_2_' is the fact that due to no significant biomass increase during the adaptation pulses, the carbon dioxide evolution rate (CER), which can easily be derived in online mode, can be used to determine Δtime_adapt_, as also shown in our previous study [18], and thus describes a valuable online tool for process monitoring and control. Consequently, we stuck to Δtime_adapt _as a minimum and safe parameter for complete methanol adaptation, as we have done previously [18], while the maximum of qCO_2_' should be regarded as a possible minimum prerequisite to start the fed batch feed.

#### Determination of the specific substrate uptake rates (q_s adapt _and q_s max_) using batch cultivations with repeated methanol pulses

The frequent determination of biomass and methanol concentrations allowed specific rate calculations for methanol uptake (q_s_) during the methanol pulses. Adaptation pulses of 0.5% (v/v) methanol were used to determine the parameter q_s adapt _(specific substrate uptake rate during the adaptation pulse) for each strain (Table 2). The knowledge of q_s adapt _allows the operator to adjust a specific, optimal flow of methanol during the adaptation for each single strain, thus preventing methanol accumulation.

After the methanol of the adaptation pulse was depleted, several pulses with 1% (v/v) methanol were conducted to determine the maximum specific substrate uptake rate (q_s max_) for each *P. pastoris *strain. In Figure 2, this strategy is exemplarily shown for the Mut^S ^strain KM71H, for which the specific substrate uptake rate was calculated with 0.96 ± 0.09 mmol·g^-1^·h^-1 ^in the adaptation period (q_s adapt_) and with 1.94 ± 0.19 mmol·g^-1^·h^-1 ^as a maximum during pulses (Figure 2 and Table 2). All *P. pastoris *strains were characterized with the above mentioned strategy and results are summarized in Table 2. Very different values for the single strain characteristic parameters of the different *P. pastoris *strains were determined and it becomes evident, that *P. pastoris *strains require specific conditions for an optimal adaptation to methanol and that the maximum levels of methanol uptake differ significantly between the single strains.

Summing up, our results underline the importance of analyzing different *P. pastoris *strains in more detail during methanol adaptation and regarding the maximum substrate uptake rate. By a more detailed analysis of each strain, which can be done in a fast way by the strategy described here, the bioprocess-relevant strain characteristic parameters Δtime_adapt_, q_s adapt _and q_s max _can be easily extracted and used for setting up a consecutive fed batch experiment based on q_s_. Additionally, the more detailed individual analysis of each strain during the adaptation phase delivers important information for an early process development.

### Fed batch design based on batch cultivations with methanol pulses

After characterization experiments in batch cultivations, fed batch experiments using q_s _based feeding profiles were carried out, according to our previous study [18]. Besides proving the general applicability of this feeding strategy on different *P. pastoris *strains, we wanted to check for parameter consistency, *i.e*. whether q_s max_, which had been determined in batch experiments before, could be reached in fed batch cultivations without observable methanol accumulation.

After a batch phase on glucose as substrate (volume 1.5 l), an exponential fed batch cultivation with glucose yielded in biomass concentrations of up to 70 g/l in a final volume of 2.5 l. As soon as glucose was depleted, a sample was taken to determine the current biomass concentration by measuring the OD_600 _and the DCW. Afterwards, all cultures were induced with a flow corresponding to a q_s _below q_s adapt _to guarantee a certain safety margin. The observed adaptation times during the fed batch cultivations for all strains are summarized in Table 3. All the Mut^S ^strains were characterized by slightly longer adaptation times in fed-batches compared to the batch cultivations, which might be due to the mode of providing methanol, *i.e*. in batch experiments methanol was pulsed into the reactor, resulting in a temporal excess of methanol, whereas in fed batch cultivations methanol was slowly fed into the bioreactor according to the actual biomass content, which is why it took the cells longer to fully adapt to the new substrate methanol.

**Table 3:** Results of dynamic fed batch cultivations of different *P. pastoris *strains based on q_s_.

	KM71H	KM71H HRP^a^	KM71H PDI HRP	CBS7435 HRP	SMD1168H GalOx
Δtime_adapt Fedbatch _[h]	5.5	9.7	10.4	9.2	5.3

q_s max _determined in batch experiments [mmol·g^-1^·h^-1^]	1.94 ± 0.19	2.00 ± 0.21	1.08 ± 0.10	1.54 ± 0.15	2.62 ± 0.26

q_s _reached without methanol accumulation [mmol·g^-1^·h^-1^]	1.79 ± 0.18	1.92 ± 0.19	1.22 ± 0.12	1.64 ± 0.16	2.44 ± 0.24

q_p max_^b^ [U·g^-1^·h^-1^]	-	11.0 ± 1.10	7.09 ± 0.10	6.48 ± 0.65	139 ± 13.9

^a ^data taken from [18]

^b ^representing the maximum value determined out of all q_s _periods

However, the Mut^+ ^strain SMD1168H GalOX showed a completely different behaviour: the adaptation time in the fed batch was much shorter than the one observed in the batch experiment, which might be due to the fact that the sudden excess of methanol in the batch-pulse experiment resulted in inhibition events caused by produced H_2_O_2 _and thus Δtime_adapt_, which had been determined in the batch experiment, did not really describe the time point of adaptation of the culture to methanol, but rather qCO_2_' (*vide supra*).

However, as soon as the maximum in off-gas activity was reached in the fed batch cultivations, the feed was increased to 1.0 mmol·g^-1^·h^-1 ^and then stepwise (0.5 mmol·g^-1^·h^-1^/step) every 24 hours up to q_s max_. Since q_s max _for the strain KM71H HRP PDI had been determined with just 1.08 mmol·g^-1^·h^-1^, smaller increases of 0.25 mmol·g^-1^·h^-1 ^in the q_s _steps were performed. This strategy, describing a stepwise increase of q_s _to q_s max_, was chosen to allow the detection of possible dependencies between q_s _and q_p_. At several time points during each step, samples were taken and, based on the apparent biomass content (estimated by OD_600 _measurements) feeding rates were adjusted manually corresponding to the defined q_s _set point. Since these regular adjustments of q_s _to the actual biomass content were performed, the feeding profile actually represented an accelerated exponential feeding profile, which has proven to result in higher specific productivities compared to other feeding profiles tested [18].

**Figure 3:**
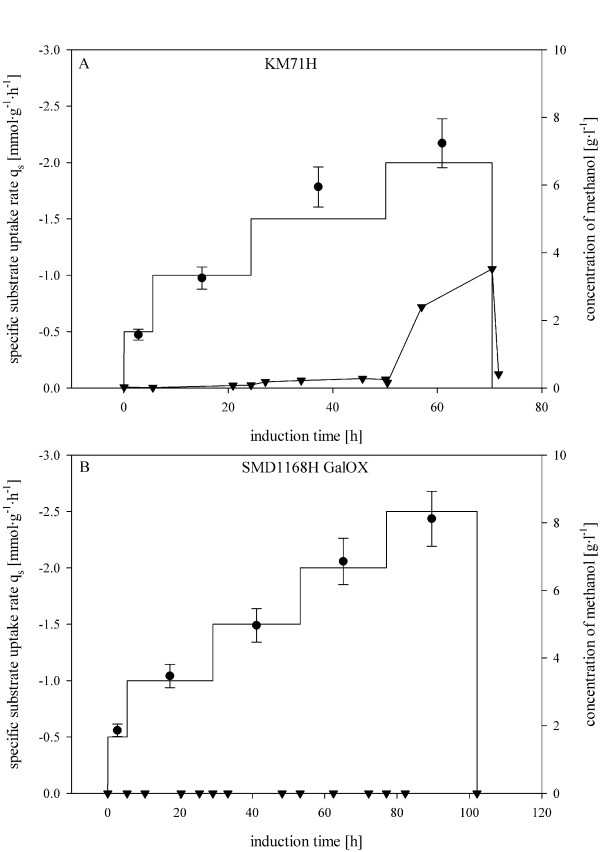
**Fed batch cultivations of different *P. pastoris *strains on methanol with a stepwise increase of q_s _to q_s max_**. A, *P. pastoris *Mut^s ^strain KM71H; B, *P. pastoris *Mut^+ ^strain SMD1186H GalOX; straight line, set point for q_s_; black dot, calculated q_s _values; black triangle, methanol concentration in the supernatant.

In Figure 3, this fed batch strategy, which was applied for all *P. pastoris *strains in this study, is exemplarily shown for the Mut^s ^strain KM71H and the Mut^+ ^strain SMD1186H GalOX. The q_s _set points were increased stepwise to a value of 2.00 mmol·g^-1^·h^-1 ^for the KM71H strain, and, as shown in Figure 3A, methanol accumulation was only observed when the feeding rate exceeded values above the respective q_s max _of 1.94 ± 0.19 mmol·g^-1^·h^-1^. When we stopped feeding after ~ 70 h, the accumulated methanol was consumed immediately. For the strain SMD1168H GalOX, the q_s _set point was stepwise increased to 2.5 mmol·g^-1^·h^-1 ^(q_s max _= 2.62 mmol·g^-1^·h^-1^) and no methanol accumulation was detected when these q_s _steps were conducted (Figure 3B). The same dynamic feeding strategy was applied to the other *P. pastoris *strains and all essential results are summarized in Table 3.

As shown in Table 3, values for q_s max_, which had been determined in batch pulsing experiments before, were reached in fed batch experiments without methanol accumulation. However, when q_s _set points were further increased, methanol accumulation was observed. This proves that the values for q_s max _from batch experiments can be found again in consecutive fed batch experiments for different *P. pastoris *strains and thus shows the great potential of this approach: by determining q_s max _in fast and easy-to-do batch experiments, the operator does not run the risk of overfeeding methanol in consecutive fed batch cultivations. However, we recommend a maximum feed flow below q_s max _to guarantee a certain safety margin.

Regarding the productivity, for all the strains, except for SMD1168H GalOX, same or even higher specific productivities were obtained in fed batch cultures compared to the batch experiments, signifying that volumetric productivities were higher in fed batch cultivations compared to batch experiments (Table 2 and Table 3). This shows that our approach employing dynamic feeding profiles [18] can be successfully applied on different *P. pastoris *strains. The lower q_p max _of the strain SMD1168H GalOX in fed batch cultivation probably resulted from copper-limitation [20], but was not investigated any further, since it was not goal of this study to optimize the production of recombinant enzymes of the single *P. pastoris *strains.

Summing up, in this study we show the general applicability of a fast approach to determine certain strain characteristic parameters, which were extracted out of batch experiments and were verified in subsequent fed batch cultures of different *P. pastoris *strains, making this approach a valuable tool for fast bioprocess development.

## Conclusions

In the present study we prove that the fast approach to determine bioprocess-relevant strain characteristic parameters and the novel dynamic feeding strategy based on q_s_, which we have described recently for one recombinant *P. pastoris *strain [18], are applicable for a variety of *P. pastoris *strains with different phenotypes producing different recombinant proteins. This underlines the great potential of this strategy as a fast and simple tool to quantify a minimal set of parameters needed to set up consecutive fed batch regimes, which is particularly important for industry, where a fast process development is essential.

Our strategy describes:

1. a batch experiment with

• a 0.5% (v/v) methanol adaptation pulse to determine Δtime_adapt _and q_s adapt_

• at least 4 consecutive 1.0% (v/v) methanol pulses to determine q_s max_

2. a dynamic fed batch feeding strategy based on q_s_, where after the adaptation of the culture to methanol (described by a maximum in off-gas activity), q_s _set points can be increased to q_s max _without observable methanol accumulation

We further show that a detailed analysis of the adaptation to methanol reveals a variability of adaptation characteristics of the different strains, highlighting that an individual analysis of potentially new strains in this respect is required to allow quantitative strain characterization and to derive parameters necessary for a consecutive fed batch set-up. The parameter Δtime_adapt _safely describes the transition condition during methanol adaptation. Since also the carbon dioxide evolution rate (CER), as well as the oxygen uptake rate (OUR), can be used to determine Δtime_adapt_, it further describes an online available data source allowing real-time monitoring and controlling of bioprocesses, which is essential under the aspect of Process Analytical Technology (PAT).

In this study we show that easy-to-do batch experiments with methanol pulses delivered valid and safe strain characteristic parameters which were consistent and precise enough to set up fed batch feeding profiles based on the specific substrate uptake rate. Our strategy is faster than the usually used continuous cultures or consecutive fed batch cultivations, and therefore allows faster process development. Besides, the strategy described here can be carried out using standard equipment without the need of cost-intensive tools; only a standard bioreactor connected to an off-gas analysis system and an HPLC or GC to determine methanol concentrations are required to carry out the experiments.

## List of Abbreviations

Δtime_adapt_: mtime for adaptation of the culture to the new substrate (methanol) [h]; μ: specific growth rate [h^-1^]; ABTS: 2, 2' azino bis 3-ethylbenzthiazoline-6-sulphonic acid; CER- carbon dioxide evolution rate [mmol·l^-1^·h^-1^]; d(qCO_2_)/dt: derivative of qCO_2_; DCW: dry cell weight; HPLC: high performance liquid chromatography; HRP: horseradish peroxidise; GalOX: galactose 6-oxidase; Mut^+^: methanol utilization phenotype; wildtype; Mut^S^: methanol utilization slow phenotype; OD_600_: optical density at 600 nm [AU]; PID: proportional-integrative-derivative controller; qCO_2_: specific carbon dioxide production rate [mmol·g^-1^·h^-1^]; qCO_2_': derivative of qCO_2_; q_p_: specific productivity [U·g^-1^·h^-1^]; q_s adapt_: specific substrate uptake rate during adaptation [mmol·g^-1^·h^-1^]; q_s max_: maximum specific substrate uptake rate [mmol·g^-1^·h^-1^]; q_s_: specific substrate uptake rate [mmol·g^-1^·h^-1^]; rpm: rounds per minute; RQ: respiratory quotient; Vvm: volume gas flow per volume liquid per minute

## Competing interests

The authors declare that they have no competing interests.

## Authors' contributions

CD designed and performed the majority of the experiments, analyzed the data and drafted the manuscript. OS designed and performed some experiments and finalized the manuscript. CH conceived the study and supervised research. All authors read and approved the final manuscript.
